# An imbalance in cluster sizes does not lead to notable loss of power in cross-sectional, stepped-wedge cluster randomised trials with a continuous outcome

**DOI:** 10.1186/s13063-017-1832-8

**Published:** 2017-03-07

**Authors:** Caroline A. Kristunas, Karen L. Smith, Laura J. Gray

**Affiliations:** 1 Diabetes Research Centre, University of Leicester, Leicester, UK; 2 Department of Health Sciences, University of Leicester, Leicester, UK

**Keywords:** Stepped wedge, Power, Sample size, Cluster randomised trial, Study design, Simulation study

## Abstract

**Background:**

The current methodology for sample size calculations for stepped-wedge cluster randomised trials (SW-CRTs) is based on the assumption of equal cluster sizes. However, as is often the case in cluster randomised trials (CRTs), the clusters in SW-CRTs are likely to vary in size, which in other designs of CRT leads to a reduction in power. The effect of an imbalance in cluster size on the power of SW-CRTs has not previously been reported, nor what an appropriate adjustment to the sample size calculation should be to allow for any imbalance. We aimed to assess the impact of an imbalance in cluster size on the power of a cross-sectional SW-CRT and recommend a method for calculating the sample size of a SW-CRT when there is an imbalance in cluster size.

**Methods:**

The effect of varying degrees of imbalance in cluster size on the power of SW-CRTs was investigated using simulations. The sample size was calculated using both the standard method and two proposed adjusted design effects (DEs), based on those suggested for CRTs with unequal cluster sizes. The data were analysed using generalised estimating equations with an exchangeable correlation matrix and robust standard errors.

**Results:**

An imbalance in cluster size was not found to have a notable effect on the power of SW-CRTs. The two proposed adjusted DEs resulted in trials that were generally considerably over-powered.

**Conclusions:**

We recommend that the standard method of sample size calculation for SW-CRTs be used, provided that the assumptions of the method hold. However, it would be beneficial to investigate, through simulation, what effect the maximum likely amount of inequality in cluster sizes would be on the power of the trial and whether any inflation of the sample size would be required.

**Electronic supplementary material:**

The online version of this article (doi:10.1186/s13063-017-1832-8) contains supplementary material, which is available to authorized users.

## Background

The stepped-wedge trial (SWT) design, also known as the ‘waiting list’ or ‘phased implementation’ design, is a relatively new trial design which is increasing in popularity [1]. A recent systematic review of SWTs published between 2010 and 2014 identified a total of 37 studies [2], whereas a previous review of SWTs published prior to January 2010 identified only 25 studies [3], of which only two were published prior to the year 2000. SWTs are, however, still a relatively rarely used design compared with others.

**Fig. 1:**
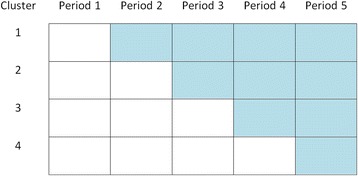
An example schematic of a stepped-wedge cluster randomised trial design. Each cell represents a data collection point. Shaded cells represent intervention periods and blank cells represent control periods

SWTs are usually cluster randomised due to the nature of the interventions that they are typically used to assess [4]. The stepped-wedge cluster randomised trial (SW-CRT) begins with no clusters in the intervention arm, and all of the clusters in the control arm [5]. Clusters are randomised to move to the intervention at prespecified times, known as steps, so that by the end of the trial all clusters are receiving the intervention [5]. One or more clusters may be randomised to switch at each time point; however, it is usual for an identical number of clusters to switch each time [5]. Measurements are obtained from each cluster between each step; they can be obtained from the same individuals each time (cohort) or from different individuals (cross-section) each time or be a mix of the two [6]. Figure 1 gives a schematic for an example SW-CRT.

There are several advantages to SW-CRTs which can make them desirable for assessing the efficacy of certain interventions. These advantages have been widely reported [1, 7, 8] and include having each cluster acting as their own control [1, 7], not withholding the intervention from a group of participants [1, 7, 8], and being able to experimentally assess the effectiveness of an intervention that for practical, logistical or financial reasons it may not be possible to assess using another design of trial [7, 8]. There are even occasions when the SW-CRT is more efficient than a parallel design, requiring a smaller sample size and fewer clusters [7]. However, there are disadvantages to SW-CRTs. Unlike a parallel design, for example, the length of a SW-CRT cannot be increased to meet recruitment targets, potentially resulting in under-powered trials. Furthermore, the analysis of SW-CRTs is complex. Hussey and Hughes [8] suggest that these studies should be analysed using generalised linear mixed models, linear mixed models or generalised estimating equations (GEEs); however, the performance of these models depends on the number of clusters, as well as whether the cluster sizes are equal or unequal [8]. These trials face the same problems as other cluster randomised trials (CRTs), with issues of unequal recruitment to clusters and the potential for entire clusters to drop out of the study. However, unlike other designs of CRTs, where sample size calculations have been developed to adjust for unequal cluster sizes, no such calculations have been proposed for use in SW-CRTs with unequal cluster sizes. In fact, the effect of an imbalance in cluster sizes on the power of SW-CRTs has yet to be reported.

### Sample size calculations for CRTs

The optimal sample size for a CRT is most often found by inflating the sample size obtained for an individually randomised trial by a design effect (DE) which accounts for the clustering [6]. For a CRT with equal cluster sizes, this is given as a function of the size of the clusters, *m*, and the intracluster correlation coefficient (ICC), *ρ* [9]:

$$ {DE}_{\mathrm{CRT}}=1+\left( m-1\right)\rho . $$

The ICC is defined as the proportion of variance accounted for by the variation between the clusters [9] and characterises the correlation between individuals from the same cluster [8]. The required sample size is found by multiplying the sample size for an individually randomised trial by the DE.

Many variations on this DE have been suggested for use in CRTs with unequal cluster sizes [10–12]. However, most of these methods require prior knowledge of the actual cluster sizes, as well as the value of the ICC; this information is usually not known until after the trial has been conducted [9]. Assuming a cluster-level analysis of a continuous outcome, Eldridge et al. [9] presented a simple DE that does not require prior knowledge of cluster sizes. This method is based on a cluster weights adjusted DE, also given by Manatunga et al. [11], and uses the mean cluster size, $\overline{m}$, and the coefficient of variation in cluster size (CV), which is the ratio of the standard deviation of cluster size to the mean cluster size. The cluster weights adjusted DE is given as:

$$ {\hat{DE}}_{\mathrm{CW}}=1+\left\{\left({CV}^2+1\right)\overline{m}-1\right\}\rho . $$

The minimum variance weights adjusted DE given by Kerry et al. [10] is not amenable to a simpler reduction in terms of the CV, and therefore requires prior knowledge of the size of the clusters. It is given as:

$$ {\widehat{DE}}_{\mathrm{MVW}}=\frac{\overline{m} I}{{\displaystyle {\sum}_{i=1}^I}\frac{m_i}{1+\left({m}_i-1\right)\rho}}, $$

where *I* is the number of clusters and *m*_*i*_ is the size of the i^th^ cluster.

### Sample size calculation for SW-CRTs

In 2013, Woertman et al. [7] derived a simple sample size formula for SW-CRTs from the formulae provided by Hussey and Hughes [8]. This formula assumes that there is no cluster by time interaction or within-subject correlation over time (i.e. cross-sectional design) and that each cluster is of an equal size. The DE derived by Woertman et al. [7] for calculating the sample size for a SW-CRT is:

$$ {DE}_{\mathrm{SW}{\textstyle \hbox{-}}\mathrm{CRT}}=\frac{1+\rho \left( k tm+ bm-1\right)}{1+\rho \left(\frac{1}{2} ktm+ bm-1\right)}.\frac{3\left(1-\rho \right)}{2 t\left( k-\frac{1}{k}\right)}, $$

where *ρ* is the ICC, *k* is the number of steps, *t* is the number of measurements taken after each step, *m* is the number of subjects within a cluster, and *b* is the number of measurements taken at baseline [7]. The required sample size for the SW-CRT is then calculated by multiplying the sample size for an individually randomised trial by the SW-CRT DE.

Although Hemming et al. [13] have recently published analytical formulae of power calculations for several variations on Hussey and Hughes’s formula [8], there is still a dearth of literature on sample size and power calculations for SWTs when compared to other designs of CRT. In particular, existing guidance focusses mainly on the cross-sectional design and assumes equality of cluster sizes, no intervention by time interaction, no cluster-by-intervention effect and categorical time effects [6].

The objective of our research was to explore possible adjustments to the DE to be used in calculating the sample size of SW-CRTs with unequal cluster sizes. We propose two adjusted DEs based on those used in CRTs and assess their appropriateness, as well as that of the Woertman et al. DE [7], by determining whether they give appropriate power under varying degrees of imbalance in cluster size.

## Methods

### Proposed design effects for SW-CRTs with unequal cluster sizes

By multiplying the sample size for an individually randomised trial by the standard DE for CRTs, and assuming equal cluster sizes, the sample size for an individually randomised trial is adjusted for the effect of clustering. The adjusted DEs make additional adjustments for the effect of an imbalance in cluster sizes. A ‘correction term’ can then be found by subtracting the standard DE from each adjusted DE. This gives the component of the DE that adjusts for the effect of an inequality in cluster size. By adding these correction terms to the standard DE for a SW-CRT, the sample size for an individually randomised trial can be adjusted for the effect of an inequality in cluster size, in addition to the effects of the clustering and stepped-wedge design:


where ${\widehat{DE}}_{\mathrm{CRT}}$ is an adjusted DE for a CRT and  is an adjusted DE for a SW-CRT.

Using the cluster and minimum variance adjusted weights DEs, given previously, we propose two adjusted DEs for SW-CRTs with unequal cluster sizes. One uses the CV in cluster size, whereas for the other, the size of each cluster must be specified. The number of subjects in each cluster in the unadjusted DE is replaced by the average cluster size, $\overline{m}$. The cluster weights adjusted DE is:


and the minimum variance weights adjusted DE is:


where *ρ* is the ICC, *k* is the number of steps, *t* is the number of measurements taken after each step, $\overline{m}$ is the average cluster size, *b* is the number of measurements taken at baseline, CV is the coefficient of variation in cluster size, *I* is the number of cluster and *m*_*i*_ is the size of the i^th^ cluster. The sample size for a SW-CRT with unequal cluster sizes can then be found by multiplying the required sample size for an individually randomised trial by one of the adjusted DEs.

### Estimating the CV in cluster size

An estimate of the CV in cluster size can be obtained by several methods, as described by Eldridge et al. [9]. This can include using previous studies, similar to the current study, to estimate the CV; however, since SWTs are a relatively new design this may be difficult. It may instead be possible to investigate and model possible sources of variation in cluster size by distinguishing between the number of individual participants in each cluster and the wider pool of individuals from which the participants are drawn [9]. The possible sources of variation can include: the distribution of the pool of individuals for each cluster; the strategies for recruiting a cluster from this population and individuals from the clusters; the patterns of response and dropout from clusters and individuals; and the distribution of eligible individuals in each cluster [9].

A more simple method of estimating the CV, when other methods are not feasible, involves using an estimate of the mean cluster size and the likely range of cluster size to give an approximation of the CV [9]. The standard deviation of cluster size is approximated by dividing the likely range of the cluster sizes by 4 [9]. The CV is then the ratio of the estimated standard deviation in cluster size to the mean cluster size.

### Simulation study

A Monte Carlo-type simulation study was conducted, using 5000 simulation runs. The unadjusted DE given by Woertman et al. [7], as well as our two proposed adjusted DEs, were used to calculate the required sample sizes for SW-CRTs with fixed power, significance level of test, effect size, ICC and number of measurements taken at each time point. Various combinations of degree of imbalance in cluster size, number of steps and average cluster size were then imposed. Data were simulated for each of these SW-CRTs using the model given by Hussey and Hughes [8] (Additional file 1), and the power to detect the true intervention effect estimated. The values of the parameters used in the simulations are given in Table 1. These values were chosen as they are commonly used in simulation studies conducted in CRTs [14–16] and are, therefore, easily transferable to SW-CRTs. Between three and eight steps were chosen after examining the results of a systematic review of SW-CRTs, which found that the majority of trials had this number of steps [3]. The cluster sizes were chosen so that they covered the range of median cluster sizes found in systematic reviews of CRTs [17–19].

**Table 1:** Parameters used during the simulation study and their values

Simulation parameter	Values
Type I error, *α*	0.05
Power, 1 − *β*	80%
ICC, *ρ*	0.05
Effect size	0.2
Average cluster size	10, 20, 30, 40
Number of steps	3, 4, 5, 6, 7, 8
Number of measurements taken at each time period	1
Imbalance in cluster size	None, moderate, Poisson, Pareto 60:40, Pareto 70:30, Pareto 80:20

To provide a focussed study on the effect of a global imbalance in cluster size on the power of SW-CRTs, the investigation was limited to cross-sectional SW-CRTs, with a continuous outcome, one measurement taken during each time period, the same number of clusters switching at each step, and no fixed time effect or delay in the effect of the intervention. We focussed on SW-CRTs where the number of individuals at each measurement period remained constant within a cluster, but where a global imbalance in the number of individuals between the clusters was introduced. The cluster sizes given are the sizes of each cluster during every measurement period. Without loss of generality, the grand mean of the response variable was set equal to 0 and the pooled variance was fixed at 1, as was used by Corrigan et al. [15] and Guittet et al. [14] in their simulation studies on CRTs. The between-cluster and within-cluster variances could then be written as *ρ* and 1 − *ρ* respectively, where *ρ* is the ICC.

Six types of imbalance in cluster size were introduced: none, moderate, Poisson, 60:40 Pareto, 70:30 Pareto and 80:20 Pareto [14]. These six methods generated varying degrees of imbalance in cluster size. When there was no imbalance in cluster size, the same number of individuals were allocated to each cluster during every time period, resulting in a CV in cluster size of 0. A moderate imbalance was introduced by, for each individual, randomly selecting with equiprobability the cluster to which they belonged at baseline and allowing the cluster size to then remain the same for the duration of the trial, creating a small imbalance in cluster size [14].

A Poisson imbalance was introduced by randomly selecting the size of each cluster from a Poisson distribution with parameter equal to the average cluster size per measurement period [14]. Individuals were then randomly allocated to a cluster [14]. If the sum of the cluster sizes was greater or less than the required sample size then individuals were randomly removed from, or added to, the clusters until the desired sample size was reached. This introduced a similar level of imbalance in cluster size to the moderate type imbalance [14].

The three Pareto type imbalances were introduced by creating two strata, one of large clusters and the other of small clusters [14]. Therefore, for an 80:20 Pareto imbalance: 80% of the individuals were assigned to the large cluster stratum, and the remaining 20% to the small cluster stratum. Twenty percent of the clusters were then assigned to the large cluster stratum, and the remaining 80% to the small cluster stratum. Within each stratum, individuals were randomly allocated to clusters so that each cluster contained the same number of individuals [14]. The range of Pareto type imbalances used in this investigation gave larger values of the CV than the other types of imbalance, thus providing a range of values of the CV in cluster size.

The CV in cluster size was estimated by running 1000 simulations for each combination of average cluster size per measurement period, number of steps and type of imbalance, and finding the mean cluster size per measurement period and standard deviation of cluster size. The CV was then calculated as the ratio of the standard deviation in cluster size to the mean cluster size per measurement period.

The required sample sizes using the standard and cluster weights DEs were calculated analytically using the estimated value of the CV for each type of imbalance in cluster size. The required sample size using the minimum variance weights adjusted DE was found by simulating a single dataset under each type of imbalance in cluster size and combination of other parameters and recording the size of each cluster at each measurement period. These cluster sizes were then used during the calculation of the DE. The CV used to calculate the minimum variance weights sample size, therefore, differs slightly from the CV for the other methods.

Analyses were conducted using GEEs with an exchangeable correlation matrix and robust standard errors. The GEE model included the response variable, treatment group and time period as covariates, and allowed for the grouping of individuals within clusters.

To examine the effect of unequal cluster sizes on the power of the SW-CRTs as the number of steps changed, the average cluster size at each measurement period was fixed at 20, whilst the number of steps was varied. To examine the effect of unequal cluster sizes on the power of the SW-CRTs as the average cluster size changed, the number of steps was fixed at four, whilst the average cluster size at each measurement period was varied.

All simulations were conducted in Stata MP 12.1. The programmes written for the simulation study are given in Additional file 2.

## Results

### Sample size calculated using the unadjusted DE, Woertman et al. [7]

#### Varying the number of steps

The Woertman et al. DE [7] was used to calculate the required sample size for SW-CRTs with average cluster size fixed at 20 and number of steps varying between three and eight. The resulting sample sizes are given in Table 2. In order to allow the same number of clusters to switch at each step, the sample size was increased by between 4.1% and 34.5%, depending on the number of steps. The actual power for these trials was, therefore, greater than the nominal 80% (Table 2). When there was no imbalance in cluster size (CV = 0), the power estimated by simulation for each trial ranged from 79.3% to 87.3% (Table 2). The actual powers, calculated by hand, are also given in Table 2. The actual power varied from the simulated power by up to 2.9 percentage points, but it has been seen elsewhere that the simulated power for CRTs will vary slightly from the actual power, even when 10,000 iterations are used [20].

**Table 2:** Design effects, sample sizes and powers for stepped-wedge cluster randomised trials (SW-CRTs) with varying average cluster size, number of steps and cluster size inequality

Average cluster size	Number of steps	DE used	Actual power (%)	Type of imbalance																						
				None (CV = 0)			Moderate				Poisson				Pareto 60:40				Pareto 70:30				Pareto 80:20			
				DE	Sample size	Power (%)	CV	DE	Sample size	Power (%)	CV	DE	Sample size	Power (%)	CV	DE	Sample size	Power (%)	CV	DE	Sample size	Power (%)	CV	DE	Sample size	Power (%)
10	4	Woertman et al.	81.8	0.535	440	81.9	0.314	0.535	440	80.1	0.320	0.535	440	81.9	0.428	0.535	440	81.7	0.909	0.538	440	80.3	1.603	0.538	440	82.0
		Cluster weights	-	0.535	440	-	0.314	0.584	480	85.5	0.320	0.586	480	84.2	0.428	0.627	520	87.7	0.909	0.948	760	95.8	1.603	1.820	1440	99.9
		Min. var. weights	-	0.535	440	-	0.317	0.568	480	85.5	0.313	0.569	480	84.2	0.420	0.593	480	84.9	0.889	0.787	640	92.2	1.622	1.362	1080	99.3
20	3	Woertman et al.	83.5	0.767	660	84.0	0.222	0.767	660	83.4	0.223	0.757	660	82.6	0.446	0.767	660	82.8	0.911	0.767	660	83.6	1.594	0.767	660	83.5
		Cluster weights	-	0.767	660	-	0.222	0.816	660	83.4	0.223	0.816	660	82.6	0.446	0.966	780	88.6	0.911	1.597	1260	97.7	1.594	3.308	2640	100.0
		Min. var. weights	-	0.767	660	-	0.222	0.790	660	83.4	0.223	0.793	660	82.6	0.405	0.844	720	87.6	0.999	1.232	1020	95.0	1.624	1.970	1560	99.2
	4	Woertman et al.	82.5	0.572	480	83.3	0.222	0.572	480	82.5	0.225	0.572	480	82.3	0.445	0.572	480	82.6	0.957	0.572	480	82.4	1.647	0.572	480	84.2
		Cluster weights	-	0.572	480	-	0.222	0.622	560	87.9	0.225	0.623	560	87.1	0.445	0.770	640	91.2	0.957	1.488	1200	99.5	1.647	3.285	2640	100.0
		Min. var. weights	-	0.572	480	-	0.201	0.592	480	82.5	0.221	0.596	480	82.3	0.450	0.670	560	88.2	0.933	0.979	800	95.3	1.557	1.789	1440	99.8
	5	Woertman et al.	83.6	0.464	400	82.0	0.221	0.464	400	84.3	0.224	0.464	400	84.0	0.444	0.464	400	83.5	0.939	0.464	400	84.0	1.689	0.464	400	84.5
		Cluster weights	-	0.464	400	-	0.221	0.512	500	89.9	0.224	0.514	500	90.5	0.444	0.661	600	94.4	0.939	1.345	1100	99.8	1.689	3.316	2700	100.0
		Min. var. weights	-	0.464	400	-	0.219	0.488	400	84.3	0.221	0.488	400	84.0	0.435	0.552	500	90.0	0.866	0.848	700	96.9	1.803	1.739	1400	100.0
	6	Woertman et al.	85.8	0.392	360	83.6	0.221	0.392	360	84.8	0.222	0.392	360	86.0	0.449	0.392	360	85.2	0.994	0.392	360	85.2	1.682	0.392	360	86.8
		Cluster weights	-	0.392	360	-	0.221	0.441	360	84.8	0.222	0.442	360	86.0	0.449	0.594	480	93.1	0.994	1.380	1200	100.0	1.682	3.221	2640	100.0
		Min. var. weights	-	0.392	360	-	0.244	0.423	360	84.8	0.229	0.416	360	86.0	0.516	0.516	480	93.1	0.977	0.823	720	100.0	1.742	1.691	1440	100.0
	7	Woertman et al.	81.7	0.341	280	79.3	0.220	0.341	280	81.5	0.222	0.341	280	81.3	0.492	0.341	280	81.1	0.971	0.341	280	82.4	1.631	0.341	280	83.4
		Cluster weights	-	0.341	280	-	0.220	0.390	420	93.3	0.222	0.391	420	93.6	0.492	0.583	560	97.9	0.971	1.284	1120	100.0	1.631	3.001	2380	100.0
		Min. var. weights	-	0.341	280	-	0.225	0.365	420	93.3	0.227	0.366	420	93.6	0.498	0.451	420	92.8	1.002	0.819	700	99.4	1.527	1.468	1260	100.0
	8	Woertman et al.	90.2	0.303	320	87.3	0.219	0.303	320	89.6	0.223	0.303	320	88.9	0.471	0.303	320	89.6	0.997	0.303	320	89.3	1.672	0.303	320	90.4
		Cluster weights	-	0.303	320	-	0.219	0.351	320	89.6	0.223	0.352	320	88.9	0.471	0.524	480	96.9	0.997	1.297	1120	100.0	1.672	3.098	2560	100.0
		Min. var. weights	-	0.303	320	-	0.239	0.328	320	89.6	0.227	0.327	320	88.9	0.482	0.411	480	96.9	1.037	0.733	640	99.4	1.646	1.536	1280	100.0
30	4	Woertman et al.	81.4	0.589	480	81.8	0.180	0.589	480	81.2	0.182	0.589	480	81.8	0.468	0.589	480	81.8	0.963	0.589	480	82.0	1.673	0.589	480	83.7
		Cluster weights	-	0.589	480	-	0.180	0.638	600	88.5	0.182	0.639	600	89.0	0.468	0.918	840	96.0	0.963	1.980	1560	99.9	1.673	4.788	3840	100.0
		Min. var. weights	-	0.589	480	-	0.168	0.605	480	81.2	0.196	0.612	600	89.0	0.467	0.706	600	88.1	0.905	1.053	840	95.8	1.676	2.158	1800	100.0
40	4	Woertman et al.	80.8	0.599	480	79.7	0.155	0.599	480	81.7	0.156	0.599	480	81.3	0.499	0.599	480	80.4	1.021	0.599	480	80.4	1.574	0.599	480	83.6
		Cluster weights	-	0.599	480	-	0.155	0.647	640	90.3	0.156	0.647	640	90.5	0.499	1.097	960	97.8	1.021	2.684	2240	100.0	1.574	5.554	4480	100.0
		Min. var. weights	-	0.599	480	-	0.141	0.610	480	81.7	0.147	0.612	640	90.5	0.416	0.703	640	89.8	1.066	1.213	960	97.5	1.763	2.249	1920	100.0

Design effects (DE) and sample sizes calculated, and power estimated, for SW-CRTs with an average cluster size of 10, 20, 30 or 40, the number of steps ranging from three to eight and increasing imbalance in cluster size, using the Woertman et al. [7] and two proposed adjusted DEs. The type I error, power, intracluster correlation coefficient (ICC) and effect size were 0.05, 80%, 0.05 and 0.2, respectively. CV, coefficient of variation in cluster size

Varying degrees of imbalance in clusters size were imposed, resulting in values of the CV in cluster size ranging from 0 to 1.689 (Table 2). Moderate and Poisson type imbalances resulted in similar, small values of the CV, which remained constant as the number of steps increased. The Pareto imbalances gave increasing values of the CV as the imbalance became more extreme and these values remained fairly constant as the number of steps increased.

**Fig. 2:**
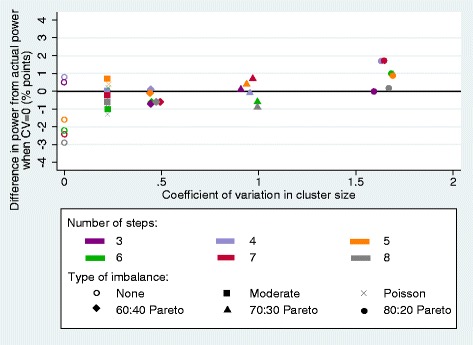
Power of stepped-wedge cluster randomised controlled trials (SW-CRTs) with varying number of steps, as variability in cluster size increases. The simulated power, relative to the analytical power, of SW-CRTs with increasing variability in cluster size, numbers of steps ranging from three to eight, average cluster size fixed at 20 and sample size calculated using the Woertman et al. design effect (DE) [7]. The type I error, power, intracluster correlation coefficient (ICC) and effect size were 0.05, 80%, 0.05 and 0.2, respectively

The varying degrees of imbalance in cluster size induced by the different types of imbalance in cluster size did not have a notable effect on the power of the SW-CRTs (Fig. 2), with the power not dropping below the actual power by any more than 1.3 percentage points. Even when the CV in cluster size was at its greatest (1.689) the power did not drop below the actual power for each trial (Table 2) and the power was often greater than the actual power. This indicated a certain amount of noise around the estimates, as has been seen elsewhere [20], and meant that a consistent pattern could not be observed.

#### Varying average cluster size

The Woertman et al. DE [7] was then used to calculate the required sample size for SW-CRTs with the number of steps fixed at four and the average cluster size varying between 10 and 40. The resulting sample sizes are given in Table 2. In order for the same number of clusters to switch at each step, the sample sizes were inflated by between 1.9% and 6.7% (Table 2). The powers estimated by simulation for these trials were between 79.7% and 83.3% when there was no imbalance in cluster size (Table 2). The actual powers, calculated by hand, varied from the simulated powers by up to 1.1 percentage points (Table 2).

Using the same six types of imbalance in cluster size, the CV took similar values, ranging from 0 to 1.673 (Table 2). For the moderate and Poisson imbalances, the CV in cluster size was seen to decrease as the average cluster size increased, whereas for the Pareto imbalances the CV was seen to increase as the average cluster size increased.

**Fig. 3:**
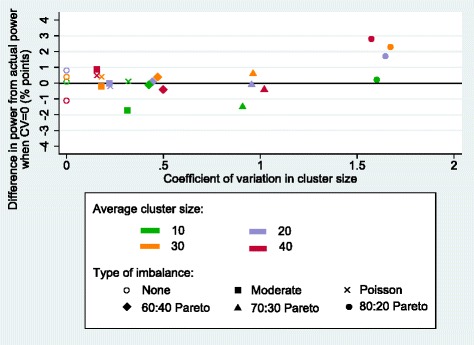
Power of stepped-wedge cluster randomised controlled trials (SW-CRTs) with varying average cluster size as variability in cluster size increases. The simulated power, relative to the analytical power, of SW-CRTs with increasing variability in cluster size, average cluster size ranging from 10 to 40, number of steps fixed at four and sample size calculated using the Woertman et al. design effect (DE) [7]. The type I error, power, intracluster correlation coefficient (ICC) and effect size were 0.05, 80%, 0.05 and 0.2, respectively

The varying degrees of imbalances in cluster size induced by the different types of imbalance in cluster size did not have a notable effect on the power of the SW-CRTs (Fig. 3). Even when the CV in cluster size was at its greatest (1.673) the power did not drop below the actual power for each trial by more than 1.7 percentage points (Table 2). Again, a certain amount of noise was observed in the estimates, as has been seen elsewhere [20], and meant that a clear pattern could not be observed.

### Sample size calculated using the two proposed adjusted DEs

When there was no imbalance in cluster size, CV = 0, both proposed adjusted DEs gave the same sample size as when the standard, Woertman et al. DE [7] was used (Table 2). This was the case for all combinations of average cluster size and number of steps that were investigated.

#### Varying the number of steps

The two proposed adjusted DEs were used to calculate the sample sizes for SW-CRTs with average cluster size fixed at 20 and number of steps varying between three and eight (Table 2). When the CV in cluster size was small (moderate or Poisson type imbalance), the sample sizes calculated using either of the proposed adjusted DEs did not increase by more than one additional cluster per step, compared to when the sample size was calculated using the Woertman et al. DE [7]. In fact, the total sample size required often remained unchanged (Table 2).

As the imbalances in cluster size became more severe, the sample sizes calculated by both of the proposed adjusted DEs varied more. Regardless of the number of steps in the SW-CRTs, or the degree of imbalance in cluster size, the minimum variance weights adjusted DE consistently gave the smaller sample size of the two proposed adjusted DEs (Table 2).

When the CV in cluster size was large, the cluster weights adjusted DEs were between 2.0 and 8.2 times greater than the Woertman et al. [7] DE, leading to total sample sizes between 1.9 and 8.5 times greater (Table 2). This resulted in severely over-powered trials (Table 2). When the most extreme imbalance in cluster size was introduced, the power of these trials reached in excess of 99%, regardless of which of the proposed adjusted DEs were used (Table 2).

#### Varying the average cluster size

The two proposed adjusted DEs were then used to calculate the sample sizes for SW-CRTs with the number of steps fixed at four and the average cluster size ranging from 10 to 40 (Table 2). When the CV in cluster size was small, the sample sizes calculated using the two proposed adjusted DEs were close to those calculated using the Woertman et al. DE [7]. Only one additional cluster was needed per step when the average cluster size was greater than 10, and two additional clusters per step were needed when the average cluster size was 10 (Table 2).

As the CV in cluster size increased, the minimum variance weights adjusted DE consistently gave sample sizes that lay between those given by the cluster weights DE and the Woertman et al. DE [7] (Table 2).

When the CV in cluster size was large, the sample sizes calculated using either the equal or cluster weights adjusted DEs were between 1.7 and 9.3 times greater than the sample sizes calculated using the Woertman et al. DE [7] (Table 2). In contrast, the minimum variance weights adjusted DE gave sample sizes that were only up to four times greater (Table 2). As the imbalances in cluster size became more extreme, both of the proposed adjusted DEs resulted in severely over-powered trials, with some attaining over 99% power for the most severe imbalances in cluster size (Table 2).

## Discussion

Sample size calculations for SW-CRTs continue to be one of the most poorly reported aspects of this trial design [2]. In those trials that do adequately describe their method of sample size calculation, there is great disparity in the methods that are being employed [2, 3]. In a recent systematic review, it was found that in some cases even the clustering of the trial had been ignored [2], and that even in those trials that did allow for clustering and the stepped-wedge design, some aspects of the design were still not taken into account [6]. For example, there is not a simple analytical calculation for determining the sample size of a cohort SW-CRTs. The sample size is, therefore, often based on a cross-sectional design, for which simple analytical sample size calculations do exist [7], which is likely to overestimate the required sample size [6].

In most SW-CRTs cluster sizes will vary to some degree and this cannot always be predicted [9]. However, there are examples of SW-CRTs where the cluster sizes were known to vary considerably prior to the trial being conducted, yet an assumption of equal cluster sizes was made when calculating the sample size [21, 22]. It is well documented that unequal cluster sizes reduce the power of CRTs [5, 9, 14, 16], yet the effect of this in SW-CRTs has not previously been reported. A loss of power can result in an under-powered study being conducted, that is likely to be unable to detect the true effect of the intervention, and would therefore be ethically dubious. Equally it is important not to run trials that are unnecessarily large. Several methods have been suggested for accounting for an inequality in cluster size when calculating the sample size for CRTs [9–11]; however, none have been suggested for use with SW-CRTs. This is the first time that the effect of unequal cluster sizes on the power of SW-CRTs has been reported and suggestions made for how to account for this when calculating the sample size.

We focussed our investigation on the effect of unequal cluster sizes on the power of a specific type of SW-CRT. The SW-CRTs that were investigated were cross-sectional, with the same number of clusters switching at each step, and assuming that there was no delay in intervention effect or effect of time. These assumptions correspond with those made by Woertman et al. [7] for their DE. Our trials had a continuous outcome and were analysed using GEEs. The results of this study are, therefore, limited to SW-CRTs of this design. A delay in intervention effect would cause the intervention effect for the groups that switch from control to intervention late in the trial to be less than for those which switch earlier. This causes a reduction in power [8]. This, as well as an imbalance in cluster size, could cause these trials to become under-powered. A similar effect would be induced by including a time effect.

We also focussed our investigation on a global imbalance in cluster sizes, where the number of individuals included in each cluster varied, but where the same number of individuals were included at each measurement period within a cluster. Another type of imbalance that may have an impact on the power of the SW-CRT would be if the number of included individuals between the different measurement periods also varied. This would be of interest for future research.

A topic that would also be of interest for future research would be to extend our research to investigate the effect of unequal cluster sizes for different values of the ICC and effect sizes. Although we focussed our investigation on SW-CRTs with an effect size of 0.2 and an ICC of 0.05, Guittet et al. [14] have shown that for parallel CRTs power decreases as the ICC increases, and although they found consistent patterns as the effect size was varied there is an impact on the power of making this change.

A strength of our investigation is our choice to simulate the values of the CV in cluster size, rather than estimating the CV analytically. For the Poisson imbalance the cluster sizes followed a Poisson distribution, with parameter the average cluster size, the CV could easily be calculated analytically by dividing the square root of the average cluster size by the average cluster size. However, in order to preserve the required sample size some individuals were added or removed from clusters during our simulations. This was done at random, with the intention of maintaining the distribution of the cluster sizes. Our simulated CVs were found to differ by no more than 0.004 from the analytical CV, demonstrating that we succeeded in preserving the correct distribution of the cluster sizes, whilst maintaining the correct sample size. The analytical calculation of the CV for the Pareto type imbalances was less straightforward. Within each strata individuals were allocated to a cluster with equiprobability. This introduced a moderate type imbalance into each strata, increasing the variability of the cluster sizes. If it were assumed that all of the clusters within a strata were of equal sizes, then the CV could easily be calculated analytically. However, this leads to an underestimation of the CV. We therefore chose to calculate the CV using simulation methods. The analytical method was found to underestimate the CV by as much as 0.189. To maintain consistency across the different types of imbalance, and to ensure that all inequality in cluster sizes was taken into account, we simulated the CV for each type of imbalance in cluster sizes and used these values in the calculation of the DE. Our results are thus truly representative of the performance of each sample size calculation method under the actual level of inequality in cluster sizes.

We have demonstrated that for the SW-CRTs investigated in this study, the sample size calculated using the Woertman et al. DE [7] provides adequate power, even when there is a large global imbalance in cluster size, with only a small loss of power (<2%) being observed. However, there was a certain degree of noise surrounding the estimated powers from the simulations and so it was difficult to distinguish a clear trend. We also stipulated that the same number of clusters must switch at each step, and therefore the sample sizes used in our investigation were typically larger than those which are often used in practice. Woertman et al. [7] state that ‘when the number of clusters that should switch at each step is not an integer, it suffices to distribute the clusters as evenly as possible over the steps’ [7]. This would lead to a smaller total sample size being required, a reduction in power, and trials that might be more sensitive to an imbalance in cluster size. The way in which the clusters are distributed over the steps may also have an effect on the power of the SW-CRT, especially if there is an imbalance in cluster size.

Further studies are needed to investigate the effect of different variations of the standard SW-CRT, on the power of these trials. Appropriate methods for sample size calculation then need to be developed to ensure that these SW-CRTs are appropriately powered, especially those using a cohort rather than cross-sectional design. In the meantime, provided that the assumptions of the method hold, the sample size calculated using the Woertman et al. DE [7] should produce an appropriately powered trial, as long as the sample size is inflated to allow the same number of clusters to switch at each step. For SW-CRTs of a nonstandard design, and when there is expected to be a substantial imbalance in cluster size, simulation methods can be used to investigate the effect of this on the power of the trial and to find the required sample size. This is in line with the recommendations made in other papers [6]. Both of our proposed DEs produced trials that were unnecessarily large and over-powered, even when there was a moderate imbalance in cluster size. We do not recommend that these DEs be used.

## Conclusion

For SW-CRTs with the same number of clusters switching at each step, a continuous outcome and analysis conducted using GEEs, even large imbalances in cluster size do not cause a notable loss of power. This is in contrast to other designs of CRT, where an imbalance in cluster size causes a significant loss of power [9, 10, 14, 16]. The standard method of sample size calculation, using the Woertman et al. DE [7] (which does not allow for unequal cluster sizes), produces trials that are appropriately powered, even when the imbalance in cluster size is large, provided that the same number of clusters switch at each step. We therefore recommend that the Woertman et al. DE [7] can be used for calculating the sample size for SW-CRT of a similar design to that which we have used during our investigation. However, it may be beneficial to researchers to consider the maximal amount of inequality in cluster size that can realistically be expected in their trial and use simulation methods to investigate the potential impact on the power and whether the sample size will need to be inflated.

For more complex designs, where the assumptions made for the Woertman et al. DE [7] do not hold, it has been recommended that simulations be used to determine the sample size required to correctly power the trial [6]. Further to this, we recommend that an inequality in cluster sizes also be considered during this process.

The implication of these findings is that many SW-CRTs that have been conducted, which assumed equal cluster sizes when calculating the sample size, may be appropriately powered, assuming that they used an appropriate method of sample size calculation, taking into account both the clustering and stepped-wedge aspects of the design. As the SW-CRT becomes more popular, further research needs to be conducted into the methodology to ensure that these trials are appropriately powered and analysed.

## Additional files

Additional file 1: Model used for data simulation. The Hussey and Hughes [8] mixed model and a simplified version corresponding to the parameters chosen for our data simulations. (DOCX 13 kb)
Additional file 2: Stata programmes. The code for running the programmes written in Stata for performing the simulation study. Programmes are given for simulating the different types of imbalance in cluster size, estimating the coefficient of variation in cluster size, calculating the total sample size required and estimating the power of the SW-CRTs. (DOCX 33 kb)

## Abbreviations

CRT: Cluster randomised trial
CV: Coefficient of variation
DE: Design effect
GEE: Generalised estimating equation
ICC: Intra-cluster correlation
SW-CRT: Stepped-wedge cluster randomised trial
SWT: Stepped-wedge trial
