# Supplementary material for: An imbalance in cluster sizes does not lead to notable loss of power in cross-sectional, stepped-wedge cluster randomised trials with a continuous outcome
Source: Trials. 2017 Mar 7;18:109. doi: 10.1186/s13063-017-1832-8 (PMC5341460; doi:10.1186/s13063-017-1832-8)
Supplement: Additional file 1: — Model used for data simulation. The Hussey and Hughes [8] mixed model and a simplified version corresponding to the parameters chosen for our data simulations. (DOCX 13 kb) [file 13063_2017_1832_MOESM1_ESM.docx]

**Model used for data simulation**

For a SW-CRT with $I$ clusters, $T$ time points, and $M$ individuals sampled per cluster per time interval, denote the response corresponding to individual $k (k$ in 1,…,$M)$ at time $j (j$ in 1,…,$T)$ from cluster $i(i$ in 1,…,$I)$ as $Y_{ijk}$. We can fit the following model for the individual responses:

$$Y_{ijk}=\mu+\alpha_{i}+\beta_{j}+X_{ij}\theta+e_{ijk}$$

where $\mu$ is the grand mean, $\alpha_{i}\sim N(0,{\sigma_{b}}^{2})$ is a random effect for cluster $i$, $\beta_{j}$ is a fixed effect for time interval $j$($j$ in 1,…,$T-1, \beta_{T}=0$ for identifiability), $X_{ij}$ is an indicator of the treatment group of cluster $i$ at time $j$ (1=intervention, 0=control), $\theta$ is the intervention effect and $e_{ijk}\sim N\left( o, \sigma_{w}^{2} \right)$ are the individual level errors, which are independently and identically distributed (IID).

For our simulations,

$$\mu=0, \beta_{j}=0, \theta=0.2$$

$${\sigma_{b}}^{2}+{\sigma_{w}}^{2}=1, {\sigma_{b}}^{2}=\rho=0.05, {\sigma_{w}}^{2}=1-\rho=0.95$$

So that the model becomes:

$$Y_{ijk}=\alpha_{i}+{0.2X}_{ij}+e_{ijk}$$

where $\alpha_{i}\sim N(0,0.05)$ is a random effect for cluster $i$, $X_{ij}$ is an indicator of the treatment group of cluster $i$ at time $j$ (1=intervention, 0=control) and $e_{ijk}\sim N\left( o, 0.95 \right)$ are the individual level errors, which are IID.
