# Supplementary material for: An imbalance in cluster sizes does not lead to notable loss of power in cross-sectional, stepped-wedge cluster randomised trials with a continuous outcome
Source: Trials. 2017 Mar 7;18:109. doi: 10.1186/s13063-017-1832-8 (PMC5341460; doi:10.1186/s13063-017-1832-8)
Supplement: Additional file 2: — Stata programmes. The code for running the programmes written in Stata for performing the simulation study. Programmes are given for simulating the different types of imbalance in cluster size, estimating the coefficient of variation in cluster size, calculating the total sample size required and estimating the power of the SW-CRTs. (DOCX 33 kb) [file 13063_2017_1832_MOESM2_ESM.docx]

STATA PROGRAMS FOR SIMULATING IMBALANCES IN CLUSTER SIZE

**No imbalance in cluster size**

n_C=number of clusters, sigb=between cluster standard deviation, ICC=value of the ICC, TREAT=treatment effect, N=average cluster size, N_S=number of clusters switching at each step, N_C=number of clusters, TN=total sample size required

*capture program drop clerrors*

*qui program define clerrors, rclass*

*syntax, n_C(int) sigb(real)*

*preserve*

*clear*

*qui set obs `n_C'*

*drawnorm u, mean(0) sds(`sigb')*

*set matsize 800*

*mkmat u, mat(u0)*

*forvalues k=1/`n_C' {*

*return scalar u0`k'=u0[`k',1]*

*}*

*end*

*capture program drop NOdatasim*

*qui program define NOdatasim, rclass*

*syntax, ICC(real) TREAT(real) N(int) N_S(int) N_C(int) TN(int)*

*clear*

*local sigb=sqrt(`icc')*

*local sigw=sqrt(1-`icc')*

*local mu1=0*

*local mu2=`treat'*

*local nw=(`n_C'/`n_S') /*number of wedges*/*

*local sizew=(`n'*`n_S')*

*local v=0*

*local nobs=`tn'*(`nw'+1)*

*local b=`sizew'*(`nw'+1)*

**set up the individual, cluster and time id variables*

*qui set obs `nobs'*

*qui gen obsnum=_n*

*qui gen byte _x = 1 if mod(obsnum[_n-1],(`nobs'/`n_C'))==0*

*qui gen byte _y = 1 if mod(obsnum[_n-1],(`nw'+1))==0*

*qui gen j = sum(_x)*

*qui replace j=j+1*

*qui gen i = sum(_y)*

*qui replace i=i+1*

*qui drop _x _y*

*qui bysort j i: gen time = _n*

*qui egen w=seq(), from(1) to (`n_S') block(`b')*

*qui label variable w "Wedge ID"*

**set up the treatment indicator variable:*

*qui gen trt=0*

*qui replace trt=1 if time>w*

** set up individual level errors:*

*drawnorm e, mean(0) sds(`sigw')*

** set up the cluster-level errors:*

*clerrors, n_C(`n_C') sigb(`sigb')*

*qui gen u0=.*

*forvalues m=1/`n_C' {*

*qui replace u0=`r(u0`m')' if j==`m'*

*}*

**generate the outcome, y:*

*local nt=(`nw'+1)*

*qui gen y=`mu1' + (`mu2'*trt) + u0 + e*

*end*

**Moderate imbalance in cluster size:**

(Uses clerrors program previously given).

n_I=total required sample size (number of individuals), n_C=number of clusters, ICC=value of the ICC, TREAT=treatment effect, N=average cluster size, N_S=number of clusters switching at each step, N_C=number of clusters, TN=total sample size required

*capture program drop moderate*

*qui program define moderate, rclass*

*syntax, n_I(int) n_C(int)*

*preserve*

*clear*

*qui set obs `n_I'*

*gen i=_n*

*gen j=floor((`n_C')*runiform()+1)*

*forvalues k=1/`n_I' {*

*return scalar clusterofindividual`k'=j[`k']*

*}*

*end*

*capture program drop MODdatasim*

*qui program define MODdatasim, rclass*

*syntax, ICC(real) TREAT(real) N(int) N_S(int) N_C(int) TN(int)*

*clear*

*local sigb=sqrt(`icc')*

*local sigw=sqrt(1-`icc')*

*local mu1=0*

*local mu2=`treat'*

*local nw=(`n_C'/`n_S')*

*local sizew=(`n'*`n_S')*

*local v=0*

*local nobs=`tn'*(`nw'+1)*

*local b=`sizew'*(`nw'+1)*

** set up individual ids:*

*qui set obs `nobs'*

*qui gen obsnum=_n*

*qui gen byte _y = 1 if mod(obsnum[_n-1],(`nw'+1))==0*

*qui gen i = sum(_y)*

*qui replace i=i+1*

*qui drop _y*

**set up cluster ids (moderately unequal) and time period id*

*moderate, n_I(`tn') n_C(`n_C')*

*qui gen j=.*

*forvalues m=1/`tn' {*

*qui replace j=`r(clusterofindividual`m')' if i==`m'*

*}*

*sort j*

*qui bysort j i : gen time = _n*

**set up the wedge id*

*gen w=1*

*forvalues k=1/`nw' {*

*replace w=`k' if ((`k'-1)*`n_S')<j & j<=(`k'*`n_S')*

*}*

*label variable w "Wedge ID"*

**set up the treatment indicator variable:*

*qui gen trt=0*

*qui replace trt=1 if time>w*

** set up individual level errors:*

*drawnorm e, mean(0) sds(`sigw')*

** set up the cluster-level errors:*

*clerrors, n_C(`n_C') sigb(`sigb')*

*qui gen u0=.*

*forvalues m=1/`n_C' {*

*qui replace u0=`r(u0`m')' if j==`m'*

*}*

**generate the outcome, y:*

*local nt=(`nw'+1)*

*qui gen y=`mu1' + (`mu2'*trt) + u0 + e*

*end*

**Poisson type imbalance in cluster size:**

(Uses clerrors program previously given).

n_I=total required sample size (number of individuals), n_C=number of clusters, N=average cluster size, ICC=value of the ICC, TREAT=treatment effect, N_S=number of clusters switching at each step, N_C=number of clusters, TN=total sample size required

*capture program drop poissonclustersizes*

*qui program define poissonclustersizes, rclass*

*syntax, n_I(int) n_C(int) N(int)*

*preserve*

**allocate a size to each cluster using Poisson distribution*

*clear*

*qui set obs `n_C'*

*qui gen j=_n*

*qui gen size=rpoisson(`n')*

*qui gen start=1*

*forvalues k=2/`n_C'{*

*qui replace start=start[`k'-1]+size[`k'-1] in `k'*

*}*

*qui gen end=sum(size)*

*local rnumber=end[`n_C']*

*local number=`rnumber'+1*

*local noI_left=`n_I'-`rnumber'*

*if `noI_left'>=0 {*

*forvalues k=1/`n_C' {*

*return scalar start`k'=start[`k']*

*return scalar end`k'=end[`k']*

*}*

*return scalar number=end[`n_C']*

*di as text "total number of individuals being used is " as result end[`n_C']*

*}*

*else {*

*local noI_over=-`noI_left'*

*local remove =`noI_over'/`n_C'*

*local removed=floor(`remove')*

*local needremoving=`noI_over'-(`removed'*`n_C')*

*qui replace size=size-`removed'*

*forvalues k=1/`needremoving'{*

*qui replace size=size-1 in `k'*

*}*

*forvalues k=2/`n_C'{*

*qui replace start=start[`k'-1]+size[`k'-1] in `k'*

*}*

*qui replace end=sum(size)*

*forvalues k=1/`n_C' {*

*return scalar start`k'=start[`k']*

*return scalar end`k'=end[`k']*

*}*

*return scalar number=end[`n_C']*

*di as text "total number of individuals being used is " as result end[`n_C']*

*}*

*end*

*capture program drop poissonSWDCRT*

*qui program define poissonSWDCRT, rclass*

*syntax, n_I(int) n_C(int) N(int)*

*qui save data, replace*

** allocate the right number of individuals to each cluster*

*clear*

*qui set obs `n_I'*

*qui gen i=_n*

*qui gen r=runiform()*

*qui sort r*

*qui gen n=_n*

*qui gen j=.*

*poissonclustersizes, n_I(`n_I') n_C(`n_C') n(`n')*

*local number=`r(number)'+1*

*local noI_left=`n_I'-`r(number)'*

*if `noI_left'>=0 {*

*forvalues k=1/`n_C' {*

*qui replace j=`k' if `r(start`k')'<=n & n<=`r(end`k')'*

*}*

*moderate, n_I(`noI_left') n_C(`n_C')*

*forvalues k=`number'/`n_I' {*

*local m=(`k'-`number'+1)*

*qui replace j=`r(clusterofindividual`m')' if n==`k'*

*}*

*}*

*else {*

*forvalues k=1/`n_C' {*

*qui replace j=`k' if `r(start`k')'<=n & n<=`r(end`k')'*

*}*

*}*

*sort i*

*forvalues k=1/`n_I' {*

*return scalar clusterofindividual`k'=j[`k']*

*}*

*use data, clear*

*end*

*capture program drop POISSONdatasim*

*qui program define POISSONdatasim, rclass*

*syntax, ICC(real) TREAT(real) N(int) N_S(int) N_C(int) TN(int)*

*clear*

*local sigb=sqrt(`icc')*

*local sigw=sqrt(1-`icc')*

*local mu1=0*

*local mu2=`treat'*

*local nw=(`n_C'/`n_S')*

*local sizew=(`n'*`n_S')*

*local v=0*

*local nobs=`tn'*(`nw'+1)*

*local b=`sizew'*(`nw'+1)*

** set up individual ids:*

*qui set obs `nobs'*

*qui gen obsnum=_n*

*qui gen byte _y = 1 if mod(obsnum[_n-1],(`nw'+1))==0*

*qui gen i = sum(_y)*

*qui replace i=i+1*

*qui drop _y*

**set up cluster ids (moderately unequal) and time period id*

*poissonSWDCRT, n_I(`tn') n_C(`n_C') n(`n')*

*qui gen j=.*

*forvalues m=1/`tn' {*

*qui replace j=`r(clusterofindividual`m')' if i==`m'*

*}*

*sort j*

*qui bysort j i : gen time = _n*

**set up the wedge id*

*gen w=1*

*forvalues k=1/`nw' {*

*replace w=`k' if ((`k'-1)*`n_S')<j & j<=(`k'*`n_S')*

*}*

*label variable w "Wedge ID"*

**set up the treatment indicator variable:*

*qui gen trt=0*

*qui replace trt=1 if time>w*

** set up individual level errors:*

*drawnorm e, mean(0) sds(`sigw')*

** set up the cluster-level errors:*

*clerrors, n_C(`n_C') sigb(`sigb')*

*qui gen u0=.*

*forvalues m=1/`n_C' {*

*qui replace u0=`r(u0`m')' if j==`m'*

*}*

**now to generate the outcome, y:*

*local nt=(`nw'+1)*

*qui gen y=`mu1' + (`mu2'*trt) + u0 + e*

*end*

**Pareto type imbalance in cluster size:**

(Uses clerrors program previously given).

n_C=number of clusters, ratio=severity of Pareto imbalance (eg ratio=0.8 for an 80:20 imbalance) n_I=total required sample size (number of individuals), ICC=value of the ICC, TREAT=treatment effect, N=average cluster size, N_S=number of clusters switching at each step, N_C=number of clusters, TN=total sample size required

*capture program drop Cpareto*

*qui program define Cpareto, rclass*

*syntax, n_C(int) ratio(real)*

*preserve*

*qui clear*

*qui set obs `n_C'*

*qui gen j=_n*

*qui gen s=floor((1)*rbinomial(1,`ratio')+1)*

*qui save par, replace*

*qui count if s==1*

*qui svret r, keep(r(N))*

*local count1=r(N)*

*qui use par, clear*

*qui count if s==2*

*qui svret r, keep(r(N))*

*local count2=r(N)*

*qui use par, clear*

*qui while (`count1'==0 | `count2'==0) {*

*qui clear*

*qui set obs `n_C'*

*qui gen j=_n*

*qui gen s=floor((1)*rbinomial(1,`ratio')+1)*

*qui save par, replace*

*qui count if s==1*

*qui svret r, keep(r(N))*

*local count1=r(N)*

*qui use par, clear*

*qui count if s==2*

*qui svret r, keep(r(N))*

*local count2=r(N)*

*qui use par, clear*

*}*

*forvalues k=1/`n_C' {*

*return scalar strata`k'=s[`k']*

*}*

*end*

*capture program drop Ipareto*

*qui program define Ipareto, rclass*

*syntax, n_I(int) ratio(real)*

*preserve*

*qui clear*

*qui set obs `n_I'*

*local r=(1-`ratio')*

*qui gen i=_n*

*qui gen s=floor((1)*rbinomial(1,`r')+1)*

*qui save parI, replace*

*qui count if s==1*

*qui svret r, keep(r(N))*

*local count1=r(N)*

*qui use parI, clear*

*qui count if s==2*

*qui svret r, keep(r(N))*

*local count2=r(N)*

*qui use parI, clear*

*qui while (`count1'==0 | `count2'==0) {*

*qui clear*

*qui set obs `n_C'*

*qui gen i=_n*

*qui gen s=floor((1)*rbinomial(1,`r')+1)*

*qui save parI, replace*

*qui count if s==1*

*qui svret r, keep(r(N))*

*local count1=r(N)*

*qui use parI, clear*

*qui count if s==2*

*qui svret r, keep(r(N))*

*local count2=r(N)*

*qui use parI, clear*

*}*

*forvalues k=1/`n_I' {*

*return scalar strata`k'=s[`k']*

*}*

*end*

*capture program drop Cstrata*

*qui program define Cstrata, rclass*

*syntax, n_C(int) ratio(real)*

*preserve*

*qui clear*

*Cpareto, n_C(`n_C') ratio(`ratio')*

**set up cluster ids*

*qui set obs `n_C'*

*qui gen j=_n*

**use the values from the program to input the strata allocations to the clusters*

*qui gen s=.*

*forvalues m=1/`n_C' {*

*qui replace s=`r(strata`m')' if j==`m'*

*return scalar Cs`m'=`r(strata`m')'*

*}*

*qui sort s*

*end*

*capture program drop Istrata*

*qui program define Istrata, rclass*

*syntax, n_I(int) ratio(real)*

*preserve*

*qui clear*

*Ipareto, n_I(`n_I') ratio(`ratio')*

**set up individual ids*

*qui set obs `n_I'*

*qui gen i=_n*

**use the values from the program to input the strata allocations to the clusters*

*qui gen s=.*

*forvalues m=1/`n_I' {*

*qui replace s=`r(strata`m')' if i==`m'*

*return scalar Is`m'=`r(strata`m')'*

*}*

*qui sort s*

*end*

*capture program drop pareto*

*qui program define pareto, rclass*

*syntax, n_I(int) n_C(int) ratio(real)*

*qui save data, replace*

**allocate individuals to strata*

*qui clear*

*qui set obs `n_I'*

*qui gen i=_n*

*Istrata, n_I(`n_I') ratio(`ratio')*

*qui gen Is=.*

*forvalues k=1/`n_I' {*

*qui replace Is=`r(Is`k')' if i==`k'*

*}*

*qui save Istrata, replace*

*preserve*

*forval k=1/2 {*

*qui keep if Is==`k'*

*qui save Istrata`k', replace*

*restore, preserve*

*}*

**allocate clusters to strata*

*restore, not*

*qui clear*

*qui set obs `n_C'*

*qui gen j=_n*

*Cstrata, n_C(`n_C') ratio(`ratio')*

*qui gen Cs=.*

*forvalues k=1/`n_C' {*

*qui replace Cs=`r(Cs`k')' if j==`k'*

*}*

*qui save Cstrata, replace*

*preserve*

*forval k=1/2 {*

*qui keep if Cs==`k'*

*qui save Cstrata`k', replace*

*restore, preserve*

*}*

*restore, not*

**find number of clusters per strata and number of individuals per strata*

*use Cstrata1, clear*

*qui gen cn=_n*

*qui count if Cs==1*

*qui drop Cs*

*qui save Cstrata1, replace*

*qui clear*

*qui set obs 1*

*qui gen n_C1=`r(N)'*

*local n_C1=`r(N)'*

*preserve*

*qui use Cstrata2, clear*

*qui gen cn=_n*

*qui count if Cs==2*

*qui drop Cs*

*qui save Cstrata2, replace*

*restore*

*qui gen n_C2=`r(N)'*

*local n_C2=`r(N)'*

*qui use Istrata1, clear*

*qui count if Is==1*

*qui clear*

*qui set obs 1*

*qui gen n_I1=`r(N)'*

*local n_I1=`r(N)'*

*preserve*

*qui use Istrata2, clear*

*qui count if Is==2*

*restore*

*qui gen n_I2=`r(N)'*

*local n_I2=`r(N)'*

**use these numbers to assign individuals in each strata to a cluster in their strata*

*qui use Istrata1, clear*

*local split floor(`n_I1'/`n_C1')*

*qui gen rand=runiform()*

*qui sort rand*

*qui gen n=_n*

*qui gen byte _x = 1 if mod(n[_n-1],(`split'))==0*

*qui gen cn = sum(_x)*

*qui replace cn=cn+1*

*qui count if cn>`n_C1'*

*local over=`r(N)'*

*moderate, n_I(`over') n_C(`n_C1')*

*forvalues k=1/`over' {*

*local m=`k'+(`split'*`n_C1')*

*qui replace cn=`r(clusterofindividual`k')' if n==`m'*

*}*

*qui merge m:1 cn using Cstrata1*

*forvalues k=1/`n_I1' {*

*local i`k'=i[`k']*

*return scalar clusterofindividual`i`k''=j[`k']*

*}*

*qui use Istrata2, clear*

*local split floor(`n_I2'/`n_C2')*

*qui gen rand=runiform()*

*qui sort rand*

*qui gen n=_n*

*qui gen byte _x = 1 if mod(n[_n-1],(`split'))==0*

*qui gen cn = sum(_x)*

*qui replace cn=cn+1*

*qui count if cn>`n_C2'*

*local over=`r(N)'*

*moderate, n_I(`over') n_C(`n_C2')*

*forvalues k=1/`over' {*

*local m=`k'+(`split'*`n_C2')*

*qui replace cn=`r(clusterofindividual`k')' if n==`m'*

*}*

*qui merge m:1 cn using Cstrata2*

*forvalues k=1/`n_I2' {*

*local i`k'=i[`k']*

*return scalar clusterofindividual`i`k''=j[`k']*

*}*

*qui use data, clear*

*end*

*capture program drop PARETOdatasim*

*qui program define PARETOdatasim, rclass*

*syntax, ICC(real) TREAT(real) N(int) N_S(int) N_C(int) TN(int) ratio(real)*

*clear*

*local sigb=sqrt(`icc')*

*local sigw=sqrt(1-`icc')*

*local mu1=0*

*local mu2=`treat'*

*local nw=(`n_C'/`n_S')*

*local sizew=(`n'*`n_S')*

*local v=0*

*local nobs=`tn'*(`nw'+1)*

*local b=`sizew'*(`nw'+1)*

** set up individual ids:*

*qui set obs `nobs'*

*qui gen obsnum=_n*

*qui gen byte _y = 1 if mod(obsnum[_n-1],(`nw'+1))==0*

*qui gen i = sum(_y)*

*qui replace i=i+1*

*qui drop _y*

**set up cluster ids (moderately unequal) and time period id*

*pareto, n_I(`tn') n_C(`n_C') ratio(`ratio')*

*qui gen j=.*

*forvalues m=1/`tn' {*

*qui replace j=`r(clusterofindividual`m')' if i==`m'*

*}*

*sort j*

*qui bysort j i : gen time = _n*

**set up the wedge id*

*gen w=1*

*forvalues k=1/`nw' {*

*replace w=`k' if ((`k'-1)*`n_S')<j & j<=(`k'*`n_S')*

*}*

*label variable w "Wedge ID"*

**set up the treatment indicator variable:*

*qui gen trt=0*

*qui replace trt=1 if time>w*

** set up individual level errors:*

*drawnorm e, mean(0) sds(`sigw')*

** set up the cluster-level errors:*

*clerrors, n_C(`n_C') sigb(`sigb')*

*qui gen u0=.*

*forvalues m=1/`n_C' {*

*qui replace u0=`r(u0`m')' if j==`m'*

*}*

**generate the outcome, y:*

*local nt=(`nw'+1)*

*qui gen y=`mu1' + (`mu2'*trt) + u0 + e*

*end*

STATA PROGRAMS FOR ESTIMATING THE COEFFICIENT OF VARIATION IN CLUSTER SIZE

N_C=number of clusters, M=number of measurements taken at each step multiplied by the number of steps+1,

*capture program drop size*

*qui program define size, rclass*

*syntax, N_C(int) M(int)*

*qui save data, replace*

*forvalues k=1/`n_C' {*

*qui count if j==`k'*

*qui svret r, keep(r(N))*

*qui local Csize`k'=r_N/`m'*

*return scalar Csize`k'=`Csize`k''*

*qui use data, clear*

*}*

*end*

*capture program drop cv*

*qui program define cv, rclass*

*syntax, N_C(int) M(int)*

*qui save data, replace*

*qui clear*

*qui set obs `n_C'*

*qui gen j=_n*

*qui gen size=.*

*qui save sizes, replace*

*qui use data, clear*

*size, n_C(`n_C') m(`m')*

*qui use sizes, clear*

*forvalues k=1/`n_C' {*

*qui replace size=`r(Csize`k')' if j==`k'*

*}*

*qui sum size*

*svret r, keep(r(mean) r(sd))*

*local mean=r_mean*

*local sd=r_sd*

*local cv=`sd'/`mean'*

*return scalar mean=`mean'*

*return scalar sd=`sd'*

*return scalar cv=`cv'*

*qui use data, clear*

*di as text "The coefficient of variation in cluster size is `cv'."*

*end*

STATA PROGRAMS FOR CALCULATING THE TOTAL SAMPLE SIZE REQUIRED

ICC=value of the ICC, STEP=number of steps, TM=number of measurements taken at each time point, N=average cluster size, BM=number of measurements taken at baseline, CV=the value of the CV for different types of imbalance in cluster size (estimated from simulations)

**Standard method (assuming equal cluster sizes)**

*capture program drop standardSWDCRT*

*qui program define standardSWDCRT*

*syntax, ICC(real) STEP(int) TM(int) N(int) BM(int)*

*qui power twomeans 3 2.8, knownsds*

*svret r, keep(r(N))*

*local Nu=r_N*

*drop r_N*

*qui set obs 1*

*local DESW=((1+`icc'*(`step'*`tm'*`n'+`bm'*`n'-1))/(1+`icc'*(0.5*`step'*`tm'*`n'+`bm'* `n'-1)))*((3*(1-`icc'))/(2*`tm'*(`step'-(1/`step'))))*

*local equalN=`Nu'*`DESW'*

*local c=`equalN'/`n'*

*local s=`c'/`step'*

*gen N_S=ceil(`s')*

*gen N_C=N_S*`step'*

*gen TN=N_C*`n'*

*di as text "Assuming equal cluster sizes and same no. of cluster switching at each step,"*

*di as text "with trt effect=0.2, the total sample size required is " as result TN*

*di as text "and the number of clusters required is " as result N_C as text " with " as result N_S as text " clusters switching at each step."*

*end*

**Using cluster weights adjusted designs effects**

*capture program drop clusterweights*

*qui program define clusterweights*

*syntax, ICC(real) STEP(int) TM(int) N(int) BM(int) CV(real)*

*qui power twomeans 3 2.8, knownsds*

*svret r, keep(r(N))*

*local Nu=r_N*

*drop r_N*

*preserve*

*clear*

*qui set obs 1*

*local SWDE=((1+`icc'*(`step'*`tm'*`n'+`bm'*`n'-1))/(1+`icc'*(0.5*`step'*`tm'*`n'+`bm'* `n'-1)))*((3*(1-`icc'))/(2*`tm'*(`step'-(1/`step'))))*

*local DEue=(`cv'*`cv'*`n'*`icc')*

*local TDE=`SWDE'+`DEue'*

*local equalN=`Nu'*`TDE'*

**local equalN=(`Nu'*`SWDE')*(1+`DEue')*

*local c=`equalN'/`n'*

*local s=`c'/`step'*

*gen N_S=ceil(`s')*

*gen N_C=N_S*`step'*

*gen TN=N_C*`n'*

*di as text "Assuming unequal cluster sizes with cv=`cv' and same no. of cluster switching at"*

*di as text "each step, with trt effect=0.2, the total sample size required is " as result TN as text" and"*

*di as text "the number of clusters required is " as result N_C as text " with " as result N_S as text " clusters switching at each step."*

*end*

**Using minimum variance weights adjusted design effects**

*capture program drop minvarweights*

*qui program define minvarweights, rclass*

*syntax, ICC(real) STEP(int) TM(int) N(int) BM(int) n_S(int) n_C(int)*

*preserve*

*qui power twomeans 3 2.8, knownsds*

*svret r, keep(r(N))*

*local Nu=r_N*

*restore*

*local m=(`n_C'/`n_S')+1*

*forvalues k=1/`n_C' {*

*qui count if j==`k'*

*local size`k'=`r(N)'/`m'*

*}*

*clear*

*qui set obs `n_C'*

*qui gen size=.*

*forvalues k=1/`n_C' {*

*qui replace size=`size`k'' in `k'*

*}*

*qui gen y=size/(1+((size-1)*`icc'))*

*qui gen denom=sum(y)*

*local summation=denom[`n_C']*

*local num=`n'*`n_C'*

*clear*

*qui set obs 1*

*return scalar summation=`summation'*

*return scalar num=`num'*

*local SWDE=((1+`icc'*(`step'*`tm'*`n'+`bm'*`n'-1))/(1+`icc'*(0.5*`step'*`tm'*`n'+`bm'* `n'-1)))*((3*(1-`icc'))/(2*`tm'*(`step'-(1/`step'))))*

*gen DEue=(`num'/`summation') -(1+((`n'-1)*`icc'))*

*return scalar DEue=DEue*

*local TDE=`SWDE'+DEue*

*local equalN=`Nu'*`TDE'*

*local c=`equalN'/`n'*

*local s=`c'/`step'*

*gen N_S=ceil(`s')*

*gen N_C=N_S*`step'*

*gen TN=N_C*`n'*

*di as text "Assuming unequal cluster sizes with cv=`cv' and same no. of cluster switching at"*

*di as text "each step, with trt effect=0.2, the total sample size required is " as result TN as text" and"*

*di as text "the number of clusters required is " as result N_C as text " with " as result N_S as text " clusters switching at each step."*

*end*

STATA PROGRAMS TO SIMULATE ESTIMATES OF THE POWER OF STEPPED-WEDGE DESIGN CLUSTER RANDOMISED TRIALS WITH VARYING TYPES OF IMBALANCE IN CLUSTER SIZE

**No imbalance in cluster size**

(Uses programs clerrors and cv previously given).

ICC=value of the ICC, TREAT=treatment effect, N=average cluster size, N_S=number of clusters switching at each step, N_C=number of clusters, TN=total sample size required

*capture program drop NOpowersim*

*qui program define NOpowersim, rclass*

*syntax, ICC(real) TREAT(real) N(int) N_S(int) N_C(int) TN(int)*

*clear*

*local sigb=sqrt(`icc')*

*local sigw=sqrt(1-`icc')*

*local mu1=0*

*local mu2=`treat'*

*local nw=(`n_C'/`n_S')*

*local sizew=(`n'*`n_S')*

*local v=0*

*local nobs=`tn'*(`nw'+1)*

*local b=`sizew'*(`nw'+1)*

**set up the individual, cluster and time id variables*

*qui set obs `nobs'*

*qui gen obsnum=_n*

*qui gen byte _x = 1 if mod(obsnum[_n-1],(`nobs'/`n_C'))==0*

*qui gen byte _y = 1 if mod(obsnum[_n-1],(`nw'+1))==0*

*qui gen j = sum(_x)*

*qui replace j=j+1*

*qui gen i = sum(_y)*

*qui replace i=i+1*

*qui drop _x _y*

*qui bysort j i: gen time = _n*

*qui egen w=seq(), from(1) to (`n_S') block(`b')*

*qui label variable w "Wedge ID"*

**set up the treatment indicator variable:*

*qui gen trt=0*

*qui replace trt=1 if time>w*

** set up individual level errors:*

*drawnorm e, mean(0) sds(`sigw')*

** set up the cluster-level errors:*

*clerrors, n_C(`n_C') sigb(`sigb')*

*qui gen u0=.*

*forvalues m=1/`n_C' {*

*qui replace u0=`r(u0`m')' if j==`m'*

*}*

**now generate the outcome, y:*

*local nt=(`nw'+1)*

*qui gen y=`mu1' + (`mu2'*trt) + u0 + e*

**to find the value of the treatment effect for this data set:*

*quietly xtgee y trt time, i(j) t(i) robust*

*return scalar beta=_b[trt]*

*return scalar p=2*normal(-abs(_b[trt]/_se[trt]))*

**find cv:*

*local m=`nw'+1*

*cv, n_C(`n_C') m(`m')*

*return scalar cv=`r(cv)'*

*return scalar mean=`r(mean)'*

*return scalar sd=`r(sd)'*

*end*

**Moderate imbalance in cluster size**

(Uses program moderate previously given).

*capture program drop MODpowersim*

*qui program define MODpowersim, rclass*

*syntax, ICC(real) TREAT(real) N(int) N_S(int) N_C(int) TN(int)*

*clear*

*local sigb=sqrt(`icc')*

*local sigw=sqrt(1-`icc')*

*local mu1=0*

*local mu2=`treat'*

*local nw=(`n_C'/`n_S')*

*local sizew=(`n'*`n_S')*

*local v=0*

*local nobs=`tn'*(`nw'+1)*

*local b=`sizew'*(`nw'+1)*

** set up individual ids:*

*qui set obs `nobs'*

*qui gen obsnum=_n*

*qui gen byte _y = 1 if mod(obsnum[_n-1],(`nw'+1))==0*

*qui gen i = sum(_y)*

*qui replace i=i+1*

*qui drop _y*

**set up cluster ids (moderately unequal) and time period id*

*moderate, n_I(`tn') n_C(`n_C')*

*qui gen j=.*

*forvalues m=1/`tn' {*

*qui replace j=`r(clusterofindividual`m')' if i==`m'*

*}*

*sort j*

*qui bysort j i : gen time = _n*

**set up the wedge id*

*gen w=1*

*forvalues k=1/`nw' {*

*replace w=`k' if ((`k'-1)*`n_S')<j & j<=(`k'*`n_S')*

*}*

*label variable w "Wedge ID"*

**set up the treatment indicator variable:*

*qui gen trt=0*

*qui replace trt=1 if time>w*

** set up individual level errors:*

*drawnorm e, mean(0) sds(`sigw')*

** set up the cluster-level errors:*

*clerrors, n_C(`n_C') sigb(`sigb')*

*qui gen u0=.*

*forvalues m=1/`n_C' {*

*qui replace u0=`r(u0`m')' if j==`m'*

*}*

**now to generate the outcome, y:*

*local nt=(`nw'+1)*

*qui gen y=`mu1' + (`mu2'*trt) + u0 + e*

**to find the value of the treatment effect for this data set:*

*quietly xtgee y trt time, i(j) t(i) robust*

*return scalar beta=_b[trt]*

*return scalar p=2*normal(-abs(_b[trt]/_se[trt]))*

**find cv:*

*local m=`nw'+1*

*cv, n_C(`n_C') m(`m')*

*return scalar cv=`r(cv)'*

*end*

**Poisson type imbalance in cluster sizes**

(Uses poissonSWDCRT program previously given).

*capture program drop POISSONpowersim*

*qui program define POISSONpowersim, rclass*

*syntax, ICC(real) TREAT(real) N(int) N_S(int) N_C(int) TN(int)*

*clear*

*local sigb=sqrt(`icc')*

*local sigw=sqrt(1-`icc')*

*local mu1=0*

*local mu2=`treat'*

*local nw=(`n_C'/`n_S')*

*local sizew=(`n'*`n_S')*

*local v=0*

*local nobs=`tn'*(`nw'+1)*

*local b=`sizew'*(`nw'+1)*

** set up individual ids:*

*qui set obs `nobs'*

*qui gen obsnum=_n*

*qui gen byte _y = 1 if mod(obsnum[_n-1],(`nw'+1))==0*

*qui gen i = sum(_y)*

*qui replace i=i+1*

*qui drop _y*

**set up cluster ids (moderately unequal) and time period id*

*poissonSWDCRT, n_I(`tn') n_C(`n_C') n(`n')*

*qui gen j=.*

*forvalues m=1/`tn' {*

*qui replace j=`r(clusterofindividual`m')' if i==`m'*

*}*

*sort j*

*qui bysort j i : gen time = _n*

**set up the wedge id*

*gen w=1*

*forvalues k=1/`nw' {*

*replace w=`k' if ((`k'-1)*`n_S')<j & j<=(`k'*`n_S')*

*}*

*label variable w "Wedge ID"*

**set up the treatment indicator variable:*

*qui gen trt=0*

*qui replace trt=1 if time>w*

** set up individual level errors:*

*drawnorm e, mean(0) sds(`sigw')*

** set up the cluster-level errors:*

*clerrors, n_C(`n_C') sigb(`sigb')*

*qui gen u0=.*

*forvalues m=1/`n_C' {*

*qui replace u0=`r(u0`m')' if j==`m'*

*}*

**now to generate the outcome, y:*

*local nt=(`nw'+1)*

*qui gen y=`mu1' + (`mu2'*trt) + u0 + e*

**to find the value of the treatment effect for this data set:*

*quietly xtgee y trt time, i(j) t(i) robust*

*return scalar beta=_b[trt]*

*return scalar p=2*normal(-abs(_b[trt]/_se[trt]))*

**find cv:*

*local m=`nw'+1*

*cv, n_C(`n_C') m(`m')*

*return scalar cv=`r(cv)'*

*return scalar mean=`r(mean)'*

*return scalar sd=`r(sd)'*

*end*

**Pareto type imbalance in cluster sizes**

(Uses pareto program previously given), ratio= severity of Pareto imbalance (eg ratio=0.8 for an 80:20 imbalance)

*capture program drop PARETOpowersim*

*qui program define PARETOpowersim, rclass*

*syntax, ICC(real) TREAT(real) N(int) N_S(int) N_C(int) TN(int) ratio(real)*

*clear*

*local sigb=sqrt(`icc')*

*local sigw=sqrt(1-`icc')*

*local mu1=0*

*local mu2=`treat'*

*local nw=(`n_C'/`n_S')*

*local sizew=(`n'*`n_S')*

*local v=0*

*local nobs=`tn'*(`nw'+1)*

*local b=`sizew'*(`nw'+1)*

** set up individual ids:*

*qui set obs `nobs'*

*qui gen obsnum=_n*

*qui gen byte _y = 1 if mod(obsnum[_n-1],(`nw'+1))==0*

*qui gen i = sum(_y)*

*qui replace i=i+1*

*qui drop _y*

**set up cluster ids (moderately unequal) and time period id*

*pareto, n_I(`tn') n_C(`n_C') ratio(`ratio')*

*qui gen j=.*

*forvalues m=1/`tn' {*

*qui replace j=`r(clusterofindividual`m')' if i==`m'*

*}*

*sort j*

*qui bysort j i : gen time = _n*

**set up the wedge id*

*gen w=1*

*forvalues k=1/`nw' {*

*replace w=`k' if ((`k'-1)*`n_S')<j & j<=(`k'*`n_S')*

*}*

*label variable w "Wedge ID"*

**set up the treatment indicator variable:*

*qui gen trt=0*

*qui replace trt=1 if time>w*

** set up individual level errors:*

*drawnorm e, mean(0) sds(`sigw')*

** set up the cluster-level errors:*

*clerrors, n_C(`n_C') sigb(`sigb')*

*qui gen u0=.*

*forvalues m=1/`n_C' {*

*qui replace u0=`r(u0`m')' if j==`m'*

*}*

**generate the outcome, y:*

*local nt=(`nw'+1)*

*qui gen y=`mu1' + (`mu2'*trt) + u0 + e*

**to find the value of the treatment effect for this data set:*

*quietly xtgee y trt time, i(j) t(i) robust*

*return scalar beta=_b[trt]*

*return scalar p=2*normal(-abs(_b[trt]/_se[trt]))*

**find cv:*

*local m=`nw'+1*

*cv, n_C(`n_C') m(`m')*

*return scalar cv=`r(cv)'*

*return scalar mean=`r(mean)'*

*return scalar sd=`r(sd)'*

*end*
